# Application of bioactive glasses in various dental fields

**DOI:** 10.1186/s40824-022-00274-6

**Published:** 2022-07-06

**Authors:** Nazanin Jafari, Mina Seyed Habashi, Alireza Hashemi, Reza Shirazi, Nader Tanideh, Amin Tamadon

**Affiliations:** 1 Persian Gulf Marine Biotechnology Research Center, Persian Gulf Biomedical Sciences Research Institute, Bushehr University of Medical Sciences, Bushehr, Iran; 2 Department of Endodontics, School of Dentistry, Bushehr University of Medical Sciences, Bushehr, Iran; 3 Department of Anatomy, School of Medical Sciences, Medicine & Health, UNSW Sydney, Sydney, Australia; 4 Stem Cells Technology Research Center, Shiraz University of Medical Sciences, Shiraz, 71348-74478 Iran; 5 Department of Pharmacology, Medical School, Shiraz University of Medical Sciences, Shiraz, Iran; 6 PerciaVista R&D Co., Shiraz, 7514633196 Iran

**Keywords:** Bioglass, Biomedical and dental materials, Porifera, Dentistry

## Abstract

Bioactive glasses are a group of bioceramic materials that have extensive clinical applications. Their properties such as high biocompatibility, antimicrobial features, and bioactivity in the internal environment of the body have made them useful biomaterials in various fields of medicine and dentistry. There is a great variation in the main composition of these glasses and some of them whose medical usage has been approved by the US Food and Drug Administration (FDA) are called Bioglass. Bioactive glasses have appropriate biocompatibility with the body and they are similar to bone hydroxyapatite in terms of calcium and phosphate contents. Bioactive glasses are applied in different branches of dentistry like periodontics, orthodontics, endodontics, oral and maxillofacial surgery, esthetic and restorative dentistry. Also, some dental and oral care products have bioactive glasses in their compositions. Bioactive glasses have been used as dental implants in the human body in order to repair and replace damaged bones. Other applications of bioactive glasses in dentistry include their usage in periodontal disease, root canal treatments, maxillofacial surgeries, dental restorations, air abrasions, dental adhesives, enamel remineralization, and dentin hypersensitivity. Since the use of bioactive glasses in dentistry is widespread, there is a need to find methods and extensive resources to supply the required bioactive glasses. Various techniques have been identified for the production of bioactive glasses, and marine sponges have recently been considered as a rich source of it. Marine sponges are widely available and many species have been identified around the world, including the Persian Gulf. Marine sponges, as the simplest group of animals, produce different bioactive compounds that are used in a wide range of medical sciences. Numerous studies have shown the anti-tumor, anti-viral, anti-inflammatory, and antibiotic effects of these compounds. Furthermore, some species of marine sponges due to the mineral contents of their structural skeletons, which are made of biosilica, have been used for extracting bioactive glasses.

## Introduction

Over the past hundred years, investigations on materials used in dentistry have expanded dramatically [1]. Natural biomaterials such as collagen, fibrin, chitosan, hyaluronic acid, alginate, and agar as well as organic synthetic biomaterials such as polylactic acid (PLA), polyglycolic acid (PGA), poly lactide-co-glycolic acid (PLGA), and polycaprolactone (PCL), and on the other hand inorganic synthetic materials such as hydroxyapatite (HA), beta-tricalcium phosphate (β TCP) and compositions of silicate and phosphate glasses have been used in the field of dental tissue engineering [2]. Recently, new researches in the field of biomaterials have focused on tissue engineering and tissue regeneration [3]. Bioactive glass is one of the biomaterials that has revolutionized modern biomaterial-driven regenerative medicine by innovating applications in biomedicine, such as soft tissue repair and drug delivery and also, cases of its clinical applications have also been identified [4].

The first bioactive glass was invented by Larry L. Hench in 1969 [5]. According to L. Hench’s studies [6], if a substance produces a biological response that leads to a bond between the substance and tissues, it can be classified as bioactive material. Bioactive glass is based on silicate and its structure is composed of three-dimensional networks of silica when they are placed in the body they can be able to form strong chemical bonds with tissues, especially with bones [7]. Bioactive glasses dissolve when they are exposed to body fluids and then by forming the apatite crystals on their surface, they gain the ability to chemically bond with the apatite crystals which are present in bone and tooth tissues [8]. Bioactive glass has high biocompatibility and is also a type of ceramic presenting some properties of ceramics [9]. Ceramics are brittle, inorganic, and non-metallic biomaterials composed of metal-oxygen ionic bonds, and they are poor thermal conductors because they have no free electrons in their structure to transfer heat or electricity [9]. In addition, bioactive glass has several attractive properties including biocompatibility and antimicrobial properties that make it a suitable material for use as a scaffold in tissue engineering [10].

Nowadays, various biomaterials have been obtained from marine resources and attention to the seas as an accessible and natural source is increasing [11]. More than 25,000 biologically-active compounds have been identified from marine habitats [12, 13]. Marine sponges are simple invertebrate animals that are known as chemical factories in the sea because they can produce numerous different chemical compounds in water [14, 15]. Different compounds of various species of marine sponges have been studied so far [16]. Although the bioactive compounds of marine sponges show diverse chemical properties, they have great potential for application in the medical sciences [14]. The bioactive compounds of marine sponges are used in a wide range of treatments due to their antitumor, antiviral, anti-inflammatory, and antibiotic effects [17]. One of the prominent and distinguished features of the marine sponges is their ability to produce amorphous inorganic skeletal elements from hydrated silica (silica spicule) or calcium carbonate (calcareous spicule) [18]. In the skeleton of most sponges, there are silica spicules that stabilize the animal’s body structure and also play a defensive role against predators [19]. Marine sponges have been used in the production of bioactive glasses due to their mineral components such as biosilica [17, 20]. Marine sponges with silica spicules are found in the Persian Gulf [21]. These sponges can be considered as a suitable source for the production of bioactive glasses which can be used in various fields of dentistry.

## Bioactive glasses and their chemical structures

Bioactive glasses have different types according to their constituents [22]. There are many variations in the main composition of these glasses, some of them are approved by the US Food and Drug Administration (FDA) for therapeutic applications and they are known as Bioglass [4]. For example, Bioglass 45S5 and S53P4 for clinical applications are approved by the FDA [23]. Bioactive glasses have good biocompatibility properties and are similar to bone hydroxyapatite in terms of calcium and phosphate contents [24]. Bioactive glasses make it possible to bond and integrate with bone tissues by forming a layer of silica gel which stimulates the proliferation and differentiation of osteoblast cells and initiates the synthesis and deposition of organic bone matrix [25]. Therefore, bioactive glasses are widely used in medicine and dentistry [22]. For example, the first clinical application of bioactive glass was reported after applying Bioglass 45S5 for the treatment of conductive hearing loss by reconstructing the bony structures of the middle ear [26]. Up to now, more than 1.5 million people worldwide have been treated with Bioglass 45S5 [27].

There are three types of bioactive glasses, including silicate-based glass (SiO_2_), phosphate-based glass (P_2_O_5_), and borate-based glass (B_2_O_3_) [9]. The main formulation commercially is called Bioglass 45S5 which contains 45% SiO_2_, 24.5% Na_2_O, 24.5% CaO, and 6% P_2_O_5_ [28]. In addition, bioactive glasses may contain well-known biocompatible and bioactive minerals such as fluorapatite, wollastonite, diopside, and tricalcium phosphate [29, 30]. For example, an alkali-free (Na-free) bioactive glass with a formulation of 70% diopside, 10% fluorapatite, and 20% tricalcium phosphate is commercially known as FastOs BG [30]. Much more researches have been focused on changing the composition of Bioglass 45S5 by adding or removing ions to make the materials more compatible for different clinical applications [8]. Recently a novel crystallized bioactive glass-ceramic with the formulation of SiO_2_ 48.5%, Na_2_O 23.75%, CaO 23.75% and P_2_O_5_ 4.0% has been presented and is called Biosilicate which has several applications in medical sciences [31]. Table 1 shows the chemical composition of bioactive glasses.

**Table 1:** Bioactive glasses chemical composition

Bioactive glasses	SiO2	Na2O	CaO	P2O5	K_**2**_O	MgO	B_**2**_O_**3**_	Al_**2**_O_**3**_	ZnO	SrO	CaF2
**45S5**	45	24.5	24.5	6	–	–	–	–	–	–	–
**42S5**	42.1	26.3	29	2.6	–	–	–	–	–	–	–
**S53P4**	53	23	20	4	–	–	–	–	–	–	–
**55S4**	52.1	21.5	23.8	2.6	–	–	–	–	–	–	–
**58S**	60	0	36	4	–	–	–	–	–	–	–
**70S30C**	70	30	0	0	–	–	–	–	–	–	–
**45S5F**	45	24.5	12.25	6	–	–	–	–	–	–	12.5
**40S5B5**	40	24.5	24.5	6	–	–	5	–	–	–	–
**6P44**	44.2	17	18	6	4.6	10.2	–	–	–	–	–
**6P50**	49.8	15.5	15.6	6	4.2	8.9	–	–	–	–	–
**6P55**	54.5	12	15	6	4	8.5	–	–	–	–	–
**6P61**	61.1	10.3	12.6	6	2.8	7.2	–	–	–	–	–
**H12**	7.5	8	40	2.5	–	–	40	2	–	–	–
**B18**	6.5	12.5	35	1	–	–	41.5	3.5	–	–	–
**0Sr**	49.96	3.30	32.62	1.07	3.30	7.25	–	–	3	–	–
**10Sr**	49.96	3.30	29.36	1.07		7.25	–	–	3	3.26	–
**50Sr**	49.96	3.30	16.31	1.07	3.30	7.25	–	–	3	16.31	–
**100Sr**	49.96	3.30		1.07	3.30	7.25	–	–	3	32.62	–
**QM5**	41.7	5.2	36.31	4.7	1	7.82	–	–	3.13	–	–
**QM8**	41.7	5.2	30	4.7	1	14	–	–	3.13	–	–
**QM10**	41.7	5.2	26	4.7	1	18	–	–	3.13	–	–

## Application of bioactive glasses in dentistry

**Fig. 1:**
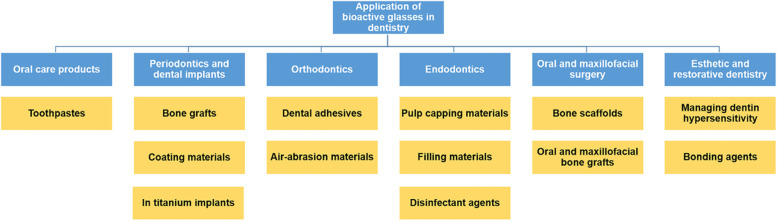
Application of bioactive glasses in dentistry

Bioactive glasses by having different advantages including having the ability to support the structure of biological tissues, being good scaffolds, and also preventing the growth of bacteria become so useful in different fields of dentistry [9]. Various applications of bioactive glasses in dentistry are mentioned in the following and briefly brought in Fig. 1. Also, some of the bioactive glasses used in dentistry are listed in Table 2.

**Table 2:** Specifications of some bioactive glasses used in dentistry

Bioactive glass brand name	properties	composition	Study type	Ref.
NovaMin	release antibacterial agents, have anti-gingivitis effect, stimulate remineralization and reduce hypersensitization	amorphous calcium sodium phosphosilicate (CSPS)/ 45% SiO2, 24.5% Na2O, 24.5% CaO and 6% P2O5/ CaNaO6PSi [32]	In vitro /Clinical trial	[33–35] [36, 37]
BiominF	Remineralization of artificial carious lesions, Dentin tubule occlusion	5% Fluorocalcium phosphosilicate bioactive glass	In vitro /Clinical trial	[38–41]
PerioGlas	grafting material in bone grafts to regenerate periodontal osseous defects	calcium phospho-silicate bioactive glass	In vitro /Clinical trial	[42–45]
QMAT3	preventing the formation of white spot lesions & stronger antimicrobial and remineralizing effects	fluoride-containing bioactive glass	In vitro	[46, 47]
45S5	removing residual orthodontic adhesive, a pulp capping material, Bio-Gutta, synthetic bone graft	45% SiO_2_, 6% P_2_O_5_, 24.5% CaO, and 24.5% Na_2_O	In vitro /Clinical trial	[24, 48]

Despite the widespread use of bioactive glass in dentistry, there are challenges to their widespread use. For example, repairing bone defects in orthopedic and dental surgery remains a major challenge. The mechanical limitations of existing glass scaffolding, along with related challenges and strategies for further improvement, need further study. In addition, emerging applications of bioactive glasses in contact with soft tissues require relative revision of biomechanical issues inorder to match the adaptation of delicate collagen tissues [49].

### Application in oral care products

Bioactive glasses have been used in various dental products especially toothpaste [33, 50]. It has been observed that bioactive glasses are useful in the formulation of toothpaste because they can release antibacterial agents, stimulate remineralization and reduce hypersensitization [24]. One of the bioactive glasses is called NovaMin which is used as an active ingredient in toothpaste to increase remineralization and reduce tooth sensitivity [51]. NovaMin (calcium-sodium-phosphate silicate) can release calcium and phosphate ions. These ions raise the pH and lead to the deposit of calcium phosphate and its conversion to hydroxyapatite [52]. NovaMin in comparison to other calcium-based products which shows an initial burst of calcium provides continuous release of calcium [53]. BiominF is another commercial product of bioactive glass which includes fluoride and phosphate and induces the formation of fluorapatite (FAP) [54]. In 2021, the first toothpaste containing bioglass and fluoride received FDA approval [55]. This toothpaste can improve acid-resistant fluorapatite on the tooth surface and inside exposed dentine tubules by controlling the release of calcium, phosphate, and fluoride ions gradually for many hours after brushing [55].

### Application in periodontics and dental implants

Periodontitis is a common chronic multifactorial inflammatory disease of the periodontium that can cause clinical attachment loss, alveolar bone loss, periodontal pocket, and gingival bleeding because of loss of periodontal tissue support [56]. This condition can also lead to alveolar bone resorption and loosening of teeth [57]. Periodontitis can cause inflammation developments around dental implants which ultimately increases the risk of implant detachment and treatment failure [58]. To improve the prognosis of dental implants, reconstruction of bone defects is essential [59]. Research on dogs has shown that bioactive glass particles have the ability to treat periodontal defects by increasing bone mineralization [60]. One of the bioactive glasses that affects bone defects is PerioGlas which has a similar formulation to Bioglass 45S5 and is widely used as a grafting material in bone grafts to regenerate periodontal osseous defects [61, 62]. PerioGlas contains 90 to 710 μm bioactive glass particles, so it can penetrate into bone defects and stimulate bone regeneration in periodontal surgeries [63, 64]. The results of bone biopsies after using PerioGlas granules as fillers in the site of tooth extraction showed new bone augmentation and confirmed good bioactivity of PerioGlas and also after a two-year clinical follow-up successful loading of the implants and evidence for implant stability were shown [65]. Also, PerioGlas reduced probing depth significantly and gained clinical attachment level (CAL) in periodontal intrabony defects [66]. So that if the amount of the harvested bone is not sufficient for the treatment of moderate to severe chronic periodontitis the mixture of autogenous bone and PerioGlas can be effective because it had similar clinical attachment gain to autogenous graft [66]. PerioGlas as a bioactive alloplast was well-tolerated by the gingival tissues [67]. Radiographs of each periodontal osseous defect and measuring of defect depth from the alveolar crest to the base of the bone defects using a Williams graduated periodontal stent demonstrated the significant improvement in bone fill when the bioactive glass is used [67].

Additionally to the bone grafting application of bioactive glasses, silica-based bioactive glasses have been used for covering implants, too [68]. The use of nanotechnology in the synthesis of bioactive glass has enhanced its application as a coating material on the surfaces of dental implants [69]. A wide range of implants are made of titanium and in some studies, bioactive glass has been used on titanium implants [70]. Covering implants with bioactive glass prevents infection and inflammation around the implants due to their antimicrobial properties [71]. The bioactive glasses increase titanium implants bond to the bone and promote their bioinert nature of them so that they reduce the total time of treatment [72–74]. In vivo, animal studies demonstrate that the titanium implants coated with bioactive glasses show significantly more osseointegration than control dental implants [75, 76]. A clinical trial was performed on 31 patients to evaluate and compare the behavior of hydroxyapatite and bioactive glass-coated implants (62 implants) in bone tissue after implantation [74]. The results showed bioactive glass coating materials were biocompatible and nontoxic and bioactive glass-coated implants were as equally successful as hydroxyapatite in achieving osseointegration and supporting final restorations. so that glass-coated implants were a viable alternative coating material for dental implants, which may allow for wider case selection criteria together with improved integration rates even in the more challenging medically compromised and osteoporotic patients [74].

### Application in orthodontics

In orthodontics, dental adhesives help to attach or bond a compound to another substance such as attachment of dental composites or orthodontic brackets to the natural tissue of the teeth [77]. The composite resin is hydrophobe and the tooth surface is hydrophile but the bonding of dental resin composite overcomes it. Thus, the adhesive acts as an interface between the two materials [77]. Adhesion of orthodontic brackets can make favorable conditions for the presence of bacteria which may lead to demineralization of the tooth and the formation of white spot lesions (WSLs) [77]. To prevent such conditions oral hygiene maintenance, regular and correct brushing, and use of fluoride toothpaste and mouthwashes are recommended [78]. Bioactive glasses have the ability to remineralize these white spot lesions [79]. Based on laboratory-based findings, the remineralization effects of bioactive glasses can be compared with topical fluoride and milk protein-derived casein phosphopeptide-amorphous calcium phosphate (CPP-ACP). These findings show that bioactive glasses enhance enamel remineralization more effectively and faster. However, clinical trials are needed to confirm their effectiveness [80]. One study found that orthodontic adhesives with bioactive glass and fluoride enhance the strength of apatite structure which may play a clinical role in preventing the formation of white spot lesions [24]. Another study found that orthodontic bonding agents containing bioactive glasses with silver or zinc elements have stronger antimicrobial and remineralizing effects compared to conventional orthodontic adhesives and the demineralization process after the pH cycling occurs at 200 to 300 μm away from orthodontic brackets [81].

The most important enamel damage due to orthodontic treatment occurs in removing the residual orthodontic adhesive after the operation. Slow-speed tungsten carbide is commonly used for this purpose [82]. QMAT3 *is a novel bioactive glass.* In one study, tungsten carbide bur, QMAT3*-air-abrasion, and* Bioglass 45S5-air-abrasion were examined in vitro to evaluate enamel damage during the processes of removing residual orthodontic adhesive. *The results show that QMAT3 bioactive glass has minimal enamel damage in comparison with* Bioglass 45S5 air abrasion *and tungsten carbide bur. Therefore, QMAT3 seems to offer a conservative approach for orthodontic adhesive removal* *[*82*]*.

### Application in endodontics

Bioactive glasses have also been used in root canal treatments [83, 84]. In dental pulp disorders, various treatment options such as pulpectomy, pulpotomy, and pulp capping are present and the materials that can be used in these treatments will play a very effective role in the prognosis of teeth and the success of the treatment [85]. In a study on rats, a novel bioactive glass was used as a pulp capping material after direct pulp capping. Then, results showed that bioactive glass stimulated the formation of heavy dentin bridges with inflammatory reactions similar to mineral trioxide aggregate (MTA) [86].

When microorganisms reach the pulp cavity, root canal treatment is prescribed in which it is necessary to use a root filler to prevent bacterial leakage as well as create a strong sealing [87, 88]. Gutta-percha in combination with Bioglass 45S5 (Bio-Gutta) can be used as an alternative to conventional gutta-percha in root canal treatments. Bio-Gutta can bond to dentin walls does not require any sealers and is also a biocompatible material [89, 90].

Also, bioactive glass can be used as a disinfectant because it has antimicrobial effects due to increasing the pH of an aqueous environment and calcium levels [91]. Bioglasses can act as topical root disinfectants in endodontics and have no effect on dentin stability [92].

### Application in oral and maxillofacial surgery

The application of bioactive glass in maxillofacial surgeries compared to other calcium phosphate compounds such as hydroxyapatite and tricalcium phosphate increases bone formation both qualitatively and quantitatively and more rapidly [93]. Bioglass was approved by the US Food and Drug Administration in 2005 as a bone stimulant [94]. Bioglass has been used as a synthetic bone graft under the commercial names Novabone in orthopedics and Perioglass in maxillofacial surgeries [95, 96]. In vitro research has shown that bioactive glass can cause bone regeneration by having effects on bone stimulation [97].

Various commercial products of bioactive glasses including Bioglass 45S5, Biogran, 70S30C bioactive glass, BonAlive, and StronBone are mainly used in oral and maxillofacial surgeries. Biogran is widely used to treat maxillofacial injuries [98]. A clinical study on about 58 cases showed that Bioglass 45S5 can be used as secondary alveolar bone grafting in patients with clefts lip and palate [99]. These procedures are commonly performed with iliac crest bone harvesting which has harvesting morbidity [99]. So using Bioglass 45S5 as an acceptable alternative to iliac crest bone harvesting can reduce harvesting morbidity and simplifies the surgery procedure [99]. One study was done on Biogran effects on volumetric changes and the new bone microarchitecture in human maxillary sinuses augmentation [100]. In this study, it was demonstrated that the addition of 50% bioactive glass to autogenous bone graft decreased the resorption volume and improved the microarchitecture of the graft [100]. Therefore, when low amounts of bone tissue are available for sinus augmentation this mixture of autogenous bone and Biogran particles seems a promising alternative to the autogenous bone only [101]. The 70S30C bioactive glass with formulations of 70% SiO_2_ and 30% CaO is effective in bone regeneration and can be used as a scaffold in bone grafting [102]. BonAlive is another type of bioactive glass is used to treat large injuries such as mandibular, orbital floor and, mastoid fractures [103, 104]. StronBone is another bioactive glass containing SrO which is used clinically to reduce bone resorption [105]. Bioactive glass can be used as a scaffold for stem cells, too. Using bioactive glass scaffolds for adipose-derived stem cells in order to treat cranio-maxillofacial hard-tissue defects at anatomically different sites, including frontal sinus, cranial bone, mandible, and nasal septum showed successful integration of the construct to the surrounding skeleton [106].

### Application in esthetic and restorative dentistry

Dentin hypersensitivity is characterized by short-term and severe toothache to thermal, chemical, or tactile stimuli. The most accepted theory for the cause of pain due to this dentin hypersensitivity is the hydrodynamic theory in which stimuli cause fluid to move in the dentinal tubules and after that, the mechanoreceptors which are near the pulp, stimulate the nerve endings of Aδ fibers resulting in sharp pain [107, 108]. According to hydrodynamic theory, dentin hypersensitivity pain can be reduced by blocking nerve endings or by sealing dentinal tubules [109, 110]. Bioactive glasses can relieve pain during dentin hypersensitivity by binding to collagen fibers and depositing hydroxyapatite in order to block dentin tubules [111]. PerioGlas tends to block dentin tubules and reduce dentin tenderness pain by bonding tightly to collagen [112].

The tooth preparation for composite restorations leads to forming a smear layer including tooth tissue debris as well as bacteria on the tooth surface. The smear layer can occlude the dentinal tubule, so it should be removed in order to enhance better bonding of the resin components. Acid-etching is performed to remove the smear layer and expose the dentinal tubules for this purpose. However, the acid-etching process activates the matrix metalloproteinases (MMPs) which destroy the collagen network of dentin and can cause microleakage [113–116]. Bonding systems containing bioactive glass in comparison with bonding systems without bioactive glass can reduce microleakages by remineralizing the mineral-deficient areas and increasing the modulus of elasticity and hardness properties at the dentin interface [117].

Biosilicate is another bioactive glass. In a clinical study, the effectiveness of Biosilicate in the treatment of dentin hypersensitivity was confirmed over a period of 6 months [31]. In fact, the particles of Biosilicate in contact with dentin reacts with the tissue inside the dentinal tubules and lead to dentinal occlusion by hydroxyapatite, thus creating a stronger bond [31]. Another study also showed that the use of suspension of Biosilicate microparticles on dentin increases the bond strength of the adhesive system [118].

## The role of complementary ions in increasing the efficiency of bioactive glasses in dentistry

Bioactive glasses have good strength, stiffness, and hardness but like other glasses, they are brittle and cannot be used in load-bearing areas [9]. Adding ions such as strontium, zinc, phosphorus, fluoride, cobalt, and silver can affect the different properties of bioactive glasses. Improving the angiogenesis with the addition of cobalt in bone grafting and increasing antimicrobial properties with the addition of silver have been observed [119, 120]. The addition of fluoride can provide numerous benefits to bioactive glasses and ceramics [121]. Fluoride decreases tooth decay by preventing demineralization of enamel and dentin and also increases remineralization and inhibits bacterial enzymes [122]. Fluoride is able to form fluorapatite (FAP) instead of carbonated hydroxyapatite and fluorapatite is more resistant to acid. Therefore, adding fluoride to bioactive glass can improve oral health [123]. Phosphate can be present as orthophosphate in bioactive glass [124]. Increasing the amount of P_2_O_5_ and other cations in fluoride-containing glasses helps to maintain network connections and increase the formation of fluorapatite [54]. This kind of bioactive glass is more desirable for clinical applications in dentistry [54]. The strontium is a bone-seeking agent similar to calcium and it is found naturally in the liver, physiological fluids, muscles, and bones [125]. The strontium-containing bioactive glass increases osteoblast proliferation and decreases osteoclast activity in cell culturing [126]. Zinc can improve the bond between glass and bone [127].

## Bioactive glass extraction from marine sponges

Considering the different applications of bioactive glass in different fields of dentistry mentioned in the previous sections, it is important to know how to obtain this material and find natural, abundant, and available sources of it. So far, various methods for extracting bioactive glass have been introduced. The melt quenching technique has been used to prepare bioactive glasses traditionally [128]. In the melt quenching process, high temperature commonly above 1000 °C is needed in order to melt ingredients, and after that rapidly quenched for freezing and fabricating the atomic structure [129]. However, the melt quenching technique provides high mechanical properties but is not able to make porous scaffolds, and also the high temperatures reduce bioactivity of the glasses [129]. Heat treatment techniques can overcome some limitations of melt quenching. For example, it can reduce thermomechanical stresses due to rapid cooling or fabricating porous scaffolds but it reduces bioactivity, too [130]. An alternative technique for bioactive glass synthesis is the sol-gel technique that uses hydrolysis and condensation reactions with low-temperature heat treatments [131]. In this way, it will be possible to produce a wide variety of glass compositions and shapes also having glasses with higher porosity [3]. Since 2006, the foam replica method has been used to produce bioactive glass scaffold that is an affordable, relatively easy, and effective technique for the development of highly porous and interconnected 3D scaffolds [132].

Natural marine sponges by having a high interconnected porous structure, the result of their evolution for 1000 years in water filtration can be used as sacrificial templates in the foam replica method to achieve superior mechanical properties [133]. Marine sponges by having various compounds such as biosilica, polyphosphate, and spongin are considered to be used in tissue engineering and reconstructive medicine [17]. Marine sponges are considered to be the earliest multicellular animals that exist at least since the late Proterozoic [134, 135]. Marine sponges are known as the members of the phylum Porifera, and they live in the oceans for about 580 million years and also more than 15, 000 species of them have been identified so far [136]. The sponges are made of an extracellular matrix containing fibrillar collagen, cells, and skeletal components, and this matrix is surrounded by a single-celled epithelial layer called pinacoderm [17]. Marine sponges have four classes and three of them, which contain more than 90% of the species, produce silica spicules. These spicules are different in the number of axis of symmetry [18]. Marine sponges naturally used biosilica for their spicule formation so that biosilica concentration is high in sponges [137]. Biosilica is enzymatically isolated from silicatein proteins of siliceous sponges [138, 139]. Sponges are the only organisms that can polymerize silica enzymatically and produce large siliceous spicules [140]. In 2021, Dudik et al. succeeded to isolated biosilica from five different Atlantic deep-sea sponges *Geodia atlantica*, *Geodia barretti*, *Stelletta normani*, *Axinella infundibuliformis*, and *Phakellia ventilabrum* [141].

In fact, the skeletons of sponges include inorganic spicules which are composed of non-crystalline hydrated amorphous silica (SiO_2_ / H_2_O) in the classes of Demospongiae, Homoscleromorpha, and Hexactinellida and calcium carbonate (CaCO_3_) in the class Calcarea [142–145]. So far, various sea sponges have been identified around the world, and a list of known species of Persian Gulf sponges is given in Table 3. As shown in Table 3, several species of the class Demospongiae and one species of the class Homoscleromorpha are present in the Persian Gulf. Nowadays, different biomaterials with osteogenic effects are demonstrated but natural-originated biomaterials compared to synthetic biomaterials are the better choice because they are more biocompatible and provide a more appropriate surface for cell attachment and growth [137, 209–211].

**Table 3:** Known species of the Persian Gulf sponges

Class	Subclass	Species	References
Calcarea	Calcaronea	*Grantia sp*	[146]
	Calcinea	*Clathrina sp*	[146]
		*Leucetta sp*	[146–150]
Demospongiae	Heteroscleromorpha	*Aaptos sp*	[151]
		*Agelas dilatata*	[152]
		*Agelas sp*	[153, 154]
		*Amphimedon viridis*	[155–157]
		*Axinella sinoxea*	[136, 158–166]
		*Callyspongia (Callyspongia) fallax*	[155]
		*Callyspongia (Callyspongia) siphonella*	[16, 150, 167, 168]
		*Callyspongia clavata*	[16, 147, 148, 167]
		*Callyspongia sp*	[148, 149, 155, 167, 169]
		*Callyspongia vasseli*	[147, 148]
		*Callyspongia sp*	[170]
		*Chalinula qatari*	[171]
		*Ciocalypta sp*	[172, 173]
		*Clathria (Microciona) mima*	[151]
		*Clathria sp*	[151, 174]
		*Cliona celata*	[175]
		*Cliona dioryssa*	[155, 169]
		*Cliona mucronata*	[176]
		*Cliona sp*	[146, 177]
		*Clionaopsis platei*	[178]
		*Cliothosa sp*	[146]
		*Dercitus (Halinastra) sp*	[176]
		*Dictyonella sp*	[179]
		*Gelliodes carnosa*	[148, 180, 181]
		*Gelliodes incrustans*	[150]
		*Gelliodes nossibea*	[182]
		*Gelliodes sp*	[146, 149, 182]
		*Gelliodes wilsoni*	[169]
		*Halichondria (Halichondria) panicea*	[151]
		*Halichondria sp*	[173, 183]
		*Haliclona*	[184]
		*Haliclona (Gellius) toxia*	[178]
		*Haliclona (Haliclona) oculata*	[185, 186]
		*Haliclona (Haliclona) simulans*	[185, 187, 188]
		*Haliclona (Haliclona) violacea*	[161, 162]
		*Haliclona (Reniera) cinerea*	[155]
		*Haliclona (Reniera) tubifera*	[161, 162]
		*Haliclona (Rhizoniera) rosea*	[155]
		*Haliclona (Soestella) caerulea*	[152, 178, 189]
		*Haliclona sp*	[21, 147, 154, 159, 169, 183, 190, 191]
		*Hemiasterella bouilloni*	[21, 183]
		*Iophon laevistylus*	[192]
		*Iophon sp*	[164]
		*Iotrochota sp*	[178]
		*Neopetrosia tuberosa*	[148]
		*Niphates furcata*	[16, 152, 167, 175, 193]
		*Niphates sp*	[21, 146, 149, 150, 154, 159, 183]
		*Pachychalina sp*	[194]
		*Pione carpenteri*	[195]
		*Pione margaritiferae*	[195]
		*Pione vastifica*	[175, 195]
		*Pseudosuberites mollis*	[169]
		*Siphonochalina sp*	[155]
		*Spheciospongia inconstans*	[182]
		*Stellettinopsis solida*	[21, 183]
		*Suberites diversicolor*	[173]
		*Suberites luna*	[171]
		*Suberites sp*	[146, 149]
		*Tedania (Tedania) sp*	[146, 149]
		*Terpios viridis*	[148]
	Keratosa	*Aplysilla sp*	[146]
		*Dictyoceratida sp*	[173]
		*Dysidea avara*	[158, 159, 161, 162, 164, 196–199]
		*Dysidea cinerea*	[147, 148]
		*Dysidea fragilis*	[151]
		*Dysidea pallescens*	[164, 193, 200]
		*Dysidea sp*	[146, 149, 150, 168, 201, 202]
		*Euryspongia sp*	[168]
		*Fascaplysinopsis reticulata*	[16, 167]
		*Hyattella sp*	[203]
		*Hyrtios erectus*	[147, 148]
		*Ircinia echinata*	[147, 148, 161, 162, 164, 182]
		*Ircinia mutans*	[161, 204–207]
		*Ircinia ramosa*	[173]
		*Ircinia sp*	[146, 151, 152, 154, 164, 178]
		*Ircinia strobilina*	[187]
		*Psammocinia sp*	[203]
		*Spongia (Spongia) arabica*	[148]
		*Spongia (Spongia) officinalis*	[151, 169]
	Verongimorpha	*Chondrilla australiensis*	[21, 178, 183, 208]
		*Chondrilla nucula*	[151, 178]
		*Chondrilla sp*	[146, 173]
		*Hexadella sp*	[146]
		*Pseudoceratina arabica*	[173]
Homoscleromorpha		*Oscarella sp*	[146]

**Fig. 2:**
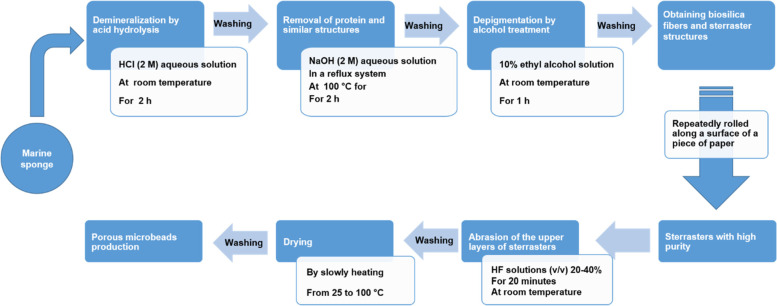
Procedure of isolation of marine sponge-derived bioactive glasses

Recently, Kaya et al. [20] extracted natural bioactive glass microspheres from spicules of marine sponge *Geodia macandrewii*. In the first step of bioactive glass extraction from the sponge, non-silicate minerals in the sponge structure should remove so that the samples are treated with HCl (2 M) aqueous solution at room temperature for 2 hours. Then the samples are washed with distilled water using Whatman filter paper to reach the neutral pH. At this stage, minerals and other similar substances are removed from the sponge samples. In the second step, the samples are placed in NaOH (2 M) aqueous solution in the reflux system at a temperature of 100 °C for 2 hours. After that, the samples are washed again with distilled water to reach a neutral pH. This basic hydrolysis method removes proteins and other similar substances. In the third step, in order to decolorize and depigmentation sponge samples are treated with 10% ethyl alcohol solution for 1 hour at room temperature to remove pigments and then washed with distilled water to reach a neutral pH. This procedure removes any pigments or similar structures that may remain in the resulting samples. After these steps, biosilica fibers and sterraster structures of sponge samples are obtained. The glass beads are placed in hydrofluoric acid solutions (v/v) 20–40% at room temperature for 20 minutes and then washed with distilled water until they reach a neutral pH. Finally, the samples are dried by gradually increasing the temperature from 25 to 100 °C. Exposure to hydrofluoric acid leads to the surface abrasion of the beads and eventually, porous biosilica beads are achieved (Fig. 2) [20]. Porous biosilica beads have been shown to be bioactive, and they form hydroxyapatite when exposed to body fluids [20]. This example clearly demonstrates that it is possible to extract bioactive glass components from marine sponges and sponges can be used as a cheap and rich natural source of bioactive glass.

## Conclusions

Since bioactive glasses have a wide range of applications in different fields of dentistry, finding an available and inexpensive resource of bioactive glass is important. Many species of marine sponges have been identified and available in the Persian Gulf which produces various types of compounds. Recent studies have shown that marine sponges can be used to produce bioactive glasses due to the presence of minerals in their structural skeletons, which are made of biosilica. Therefore, marine sponges can be scientifically and economically good choices for extracting bioactive glass. So by finding new methods and sources of bioactive glass it would be possible to enhance their applications in dentistry.

## Data Availability

Not applicable.
